# Supplementary material for: Selective molecular impairment of spontaneous neurotransmission modulates synaptic efficacy
Source: Nat Commun. 2017 Feb 10;8:14436. doi: 10.1038/ncomms14436 (PMC5311059; doi:10.1038/ncomms14436)
Supplement: Supplementary Information — Supplementary Figures. [file ncomms14436-s1.pdf]

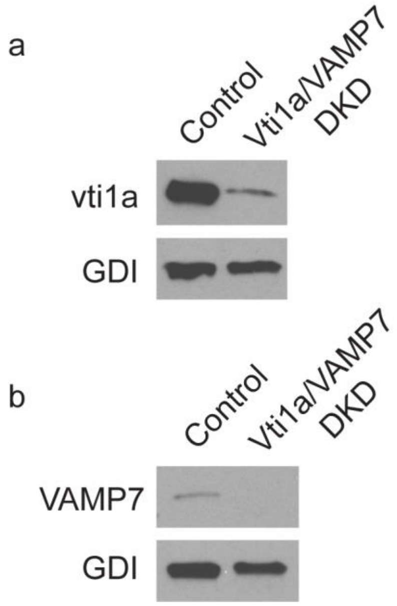

Supplementary Figure 1. Lentiviral expression of shRNA directed against both vti1a and VAMP7 in cultured rat hippocampal neurons efficiently reduces protein levels.

a. Representative immunoblot of homogenates from control and vti1a/VAMP7 DKD cultures using antibodies directed against vti1a and the loading control protein GDI. Results were replicated 3x by the laboratory. **b.** Representative immunoblot of homogenates from control and vti1a/VAMP7 DKD cultures using antibodies directed against VAMP7 and the loading control protein GDI. Results were replicated 3x by the laboratory.

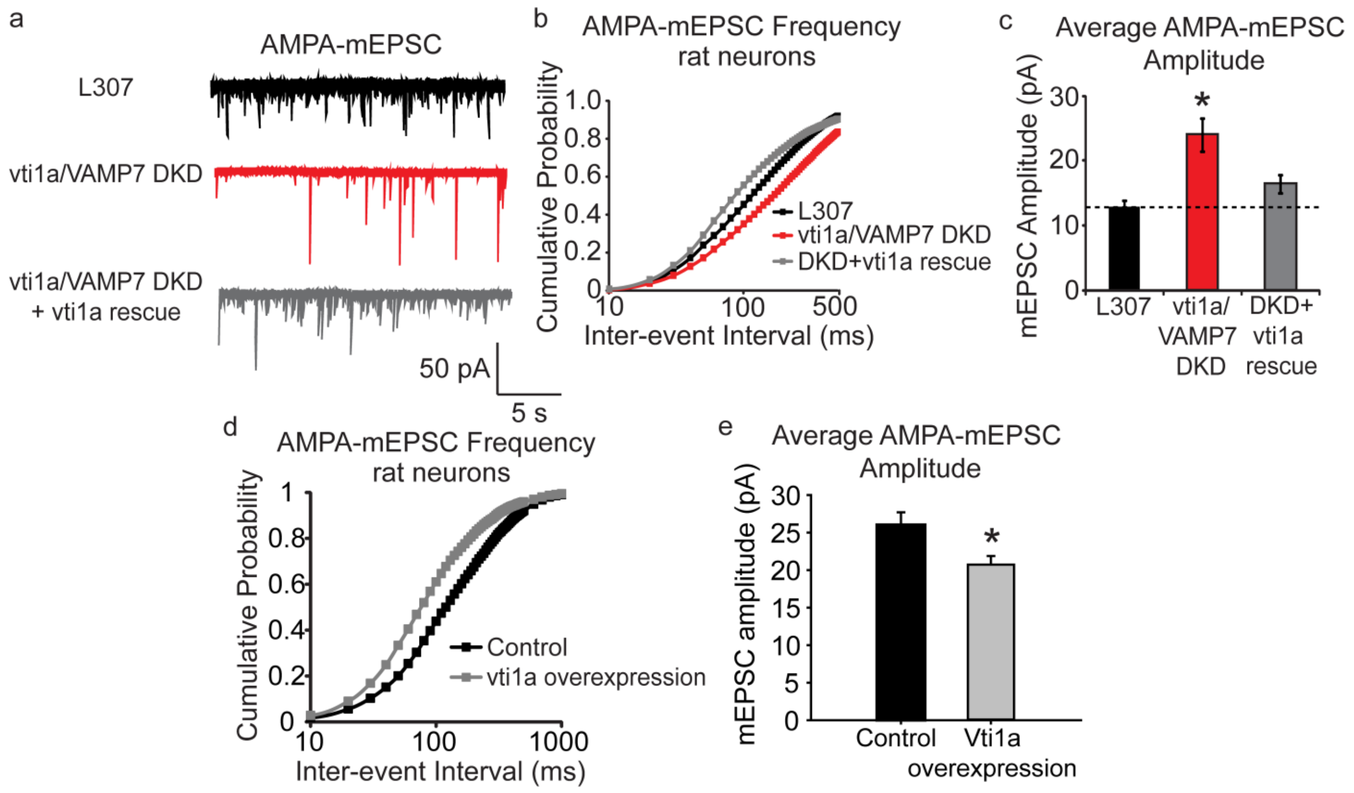

Supplementary Figure 2. ShRNA-resistant vti1a rescues AMPA-mEPSC phenotypes in cultured rat vti1a/VAMP7 DKD neurons and alters AMPA-mEPSCs in control neurons.

a. Representative traces of spontaneous AMPA event (AMPA-mEPSC) recordings in GFP control (L307), vti1a/VAMP7 DKD, and vti1a/VAMP7 DKD+vti1a-pHGFP (vti1a rescue) neurons. **b.** Cumulative probability histograms of AMPA-mEPSC inter-event intervals (L307: n=19 recordings from 7 independent cultures; vti1a/VAMP7 DKD: n=13 recordings from 5 independent cultures; vti1a/VAMP7 DKD+vti1a rescue: n=14 recordings from 4 independent cultures). Vti1a/VAMP7 DKD significantly decreased AMPA-mEPSC event frequency compared to control, but this decrease is abolished upon co-expression of shRNA-resistant vti1a-pHGFP (Kolmogorov-Smirnov test; L307 vs. vti1a/VAMP7 DKD, corrected $p<0.001$; vti1a/VAMP7 DKD vs. vti1a/VAMP7 DKD+vti1a rescue, corrected $p<0.001$; L307 vs. vti1a/VAMP7 DKD+vti1a rescue, corrected $p<0.001$). **c.** Average AMPA-mEPSC amplitudes from data analyzed in panel b. Vti1a/VAMP7 DKD significantly increased AMPA-mEPSC amplitude compared to control, and this effect was attenuated by co-expression of shRNA-resistant vti1a (L307 vs. vti1a/VAMP7 DKD, corrected $p<0.001$; L307 vs. vti1a/VAMP7 DKD+vti1a rescue, corrected $p>0.05$;

vti1a/VAMP7 DKD vs. vti1a/VAMP7 DKD+vti1a rescue, corrected $p<0.05$). **d.** Cumulative probability histograms of AMPA-mEPSC inter-event intervals (n=6 recordings from 2 independent cultures). Overexpression of vti1a in neuron cultures significantly increased the AMPA-mEPSC event frequency compared to control (Kolmogorov-Smirnov test; $p=0.0001$). **e.** Average AMPA-mEPSC amplitudes from data analyzed in panel d. Vti1a overexpression significantly decreased AMPA-mEPSC amplitude compared to control ($p=0.036$).

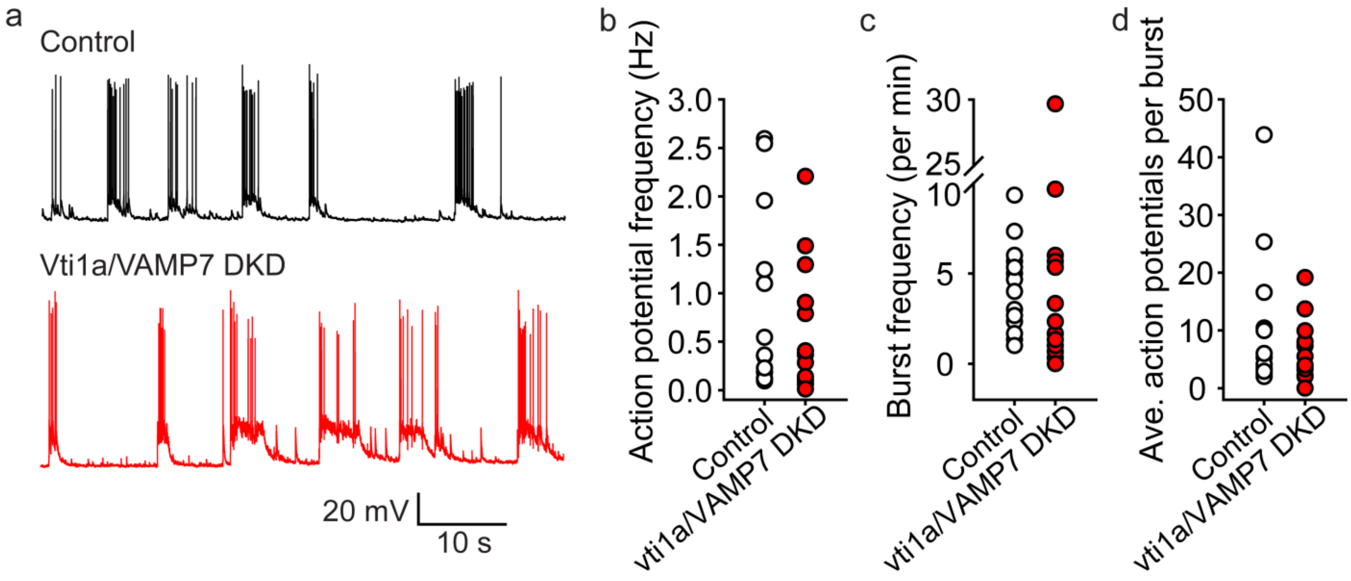

Supplementary Figure 3. Spontaneous action potential frequency is not altered in cultured neurons.

a. Representative traces of spontaneous action potentials measured in the absence of receptor blockers in current-clamp mode from control and vti1a/VAMP7 DKD neurons cultured from rat hippocampus. **b.** No differences were detected in average action potential frequency between control and vti1a/VAMP7 DKD neurons from 3 min of recording (n=14 neurons from 2 independent cultures; p=0.43). **c.** No differences were detected in average burst frequency between control and vti1a/VAMP7 DKD neurons during spontaneous activity analyzed in panel b (p=0.74). Bursts were defined as depolarizations that resulted in at least 2 action potentials before returning to resting membrane potential. **d.** No differences were detected in average number of action potentials per burst between control and vti1a/VAMP7 DKD neurons during spontaneous network activity analyzed in panel b (p=0.32).

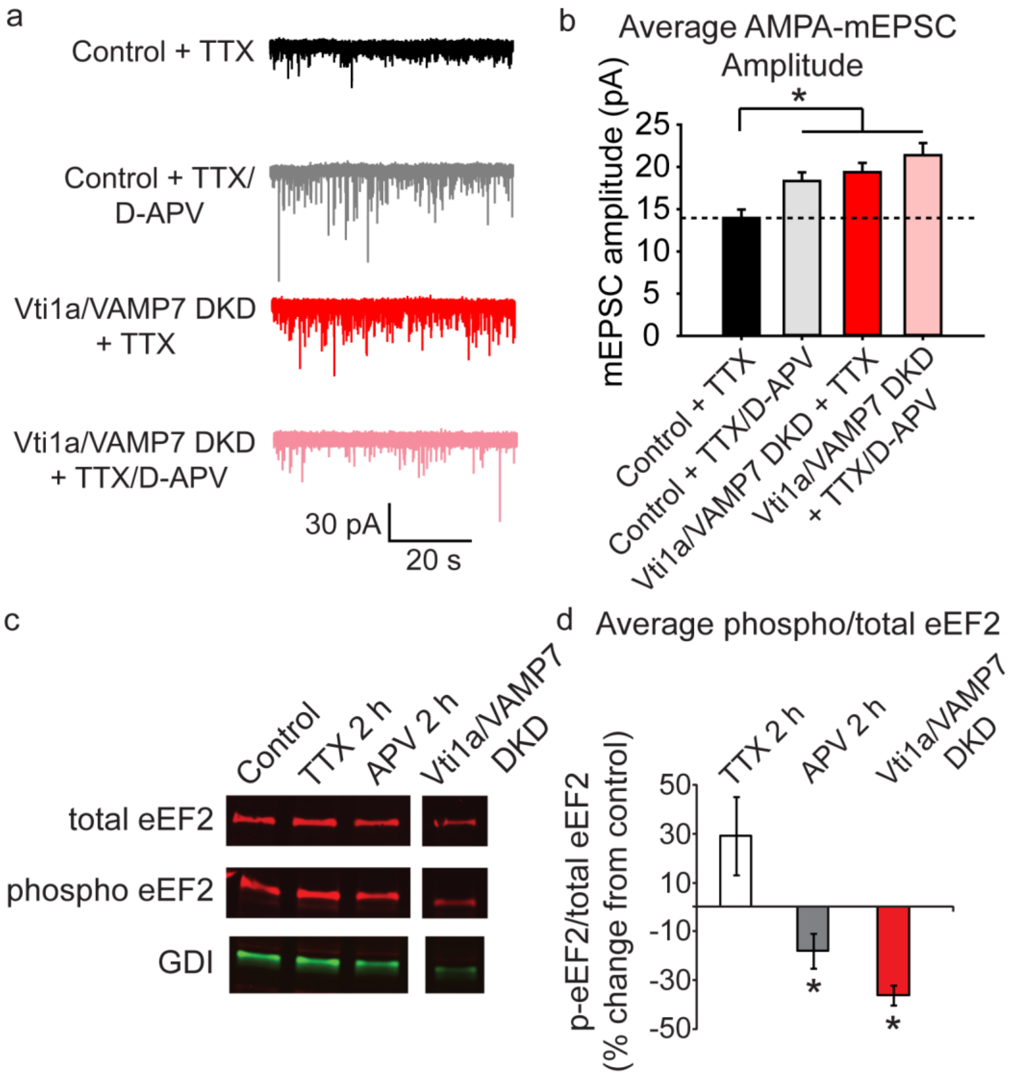

Supplementary Figure 4. Loss of vti1a and VAMP7 in cultured rat hippocampal neurons produces synaptic scaling similar to pharmacological NMDA receptor block.

a. Representative traces of AMPA-mEPSC recordings in control or vti1a/VAMP7 DKD neurons after 3 h of treatment with 2 μM TTX alone or 2 μM TTX and 50 μM D-APV. **b.** Average AMPA-mEPSC amplitudes (n=21 recordings from 4 independent cultures). A significant increase in AMPA-mEPSC amplitude was observed in control neurons treated with TTX and D-APV, vti1a/VAMP7 DKD neurons treated with TTX alone, and vti1a/VAMP7 DKD neurons treated with TTX and D-APV compared to control neurons treated with TTX alone (corrected $p<0.05$). Vti1a/VAMP7 DKD neurons treated with TTX with or without D-APV did not produce significantly different AMPA-mEPSC amplitudes

(uncorrected p=0.28). **c.** Representative immunoblot showing total eEF2, phospho-eEF2, and GDI levels in neuronal protein samples collected from control rat hippocampal neurons, control neurons treated with 1 μM TTX or 50 μM AP5 for 2 h prior to protein harvest, or vti1a/VAMP7 double knockdown neurons. A break was added where irrelevant lanes have been removed from the full Western blot image. **d.** Quantitation of phospho-eEF2 levels compared to total eEF2 levels after normalization to the loading control (control vs. TTX: n=9 independent cultures, p=0.09; control vs. AP5: n=5 independent cultures, p=0.01; control vs. vti1a/VAMP7 DKD: n=6 independent cultures, p=0.001).

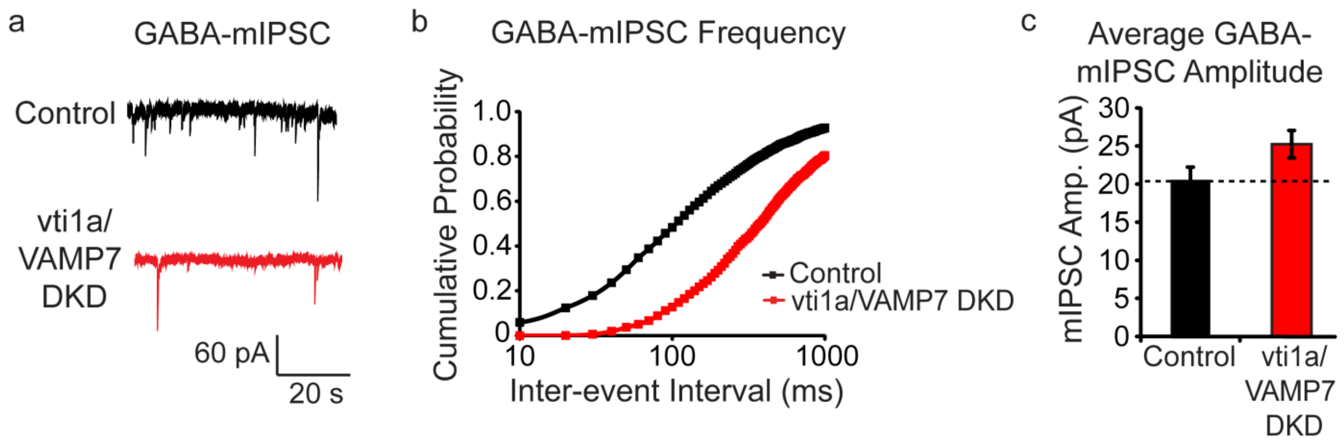

Supplementary Figure 5. Loss of vti1a and VAMP7 in cultured rat hippocampal neurons reduces GABA-mIPSC event frequency but does not significantly affect amplitudes.

a. Representative traces of spontaneous GABA event (GABA-mIPSC) recordings in control or vti1a/VAMP7 DKD neurons. **b.** Cumulative probability histograms of GABA-mIPSC inter-event intervals (control: n=9 recordings from 2 independent cultures; vti1a/VAMP7 DKD: n=8 recordings from 2 independent cultures). Vti1a/VAMP7 DKD significantly reduces GABA-mIPSC event frequency (Kolmogorov-Smirnov test; p=0.0001). **c.** Average GABA-mIPSC amplitudes from data analyzed in panel b. No significant difference was observed (p=0.12).

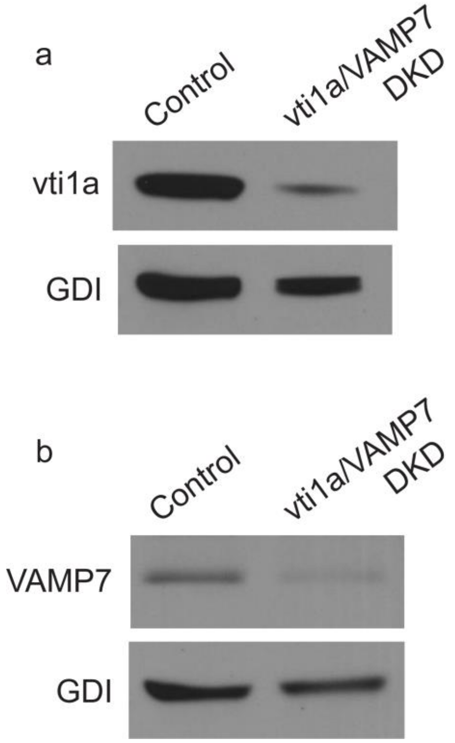

Supplementary Figure 6. AAV expression of shRNA directed against both vti1a and VAMP7 in mouse hippocampal neurons *in vivo* efficiently reduces protein levels.

a. Representative immunoblot of homogenates from control and vti1a/VAMP7 DKD hippocampi 3 weeks post-injection of AAV into dentate gyrus using antibodies directed against vti1a and the loading control protein GDI. Results were replicated 5x by the laboratory. **b.** Representative immunoblot of homogenates from control and vti1a/VAMP7 DKD hippocampi 3 weeks post-injection of AAV into dentate gyrus using antibodies directed against VAMP7 and the loading control protein GDI. Results were replicated 5x by the laboratory.

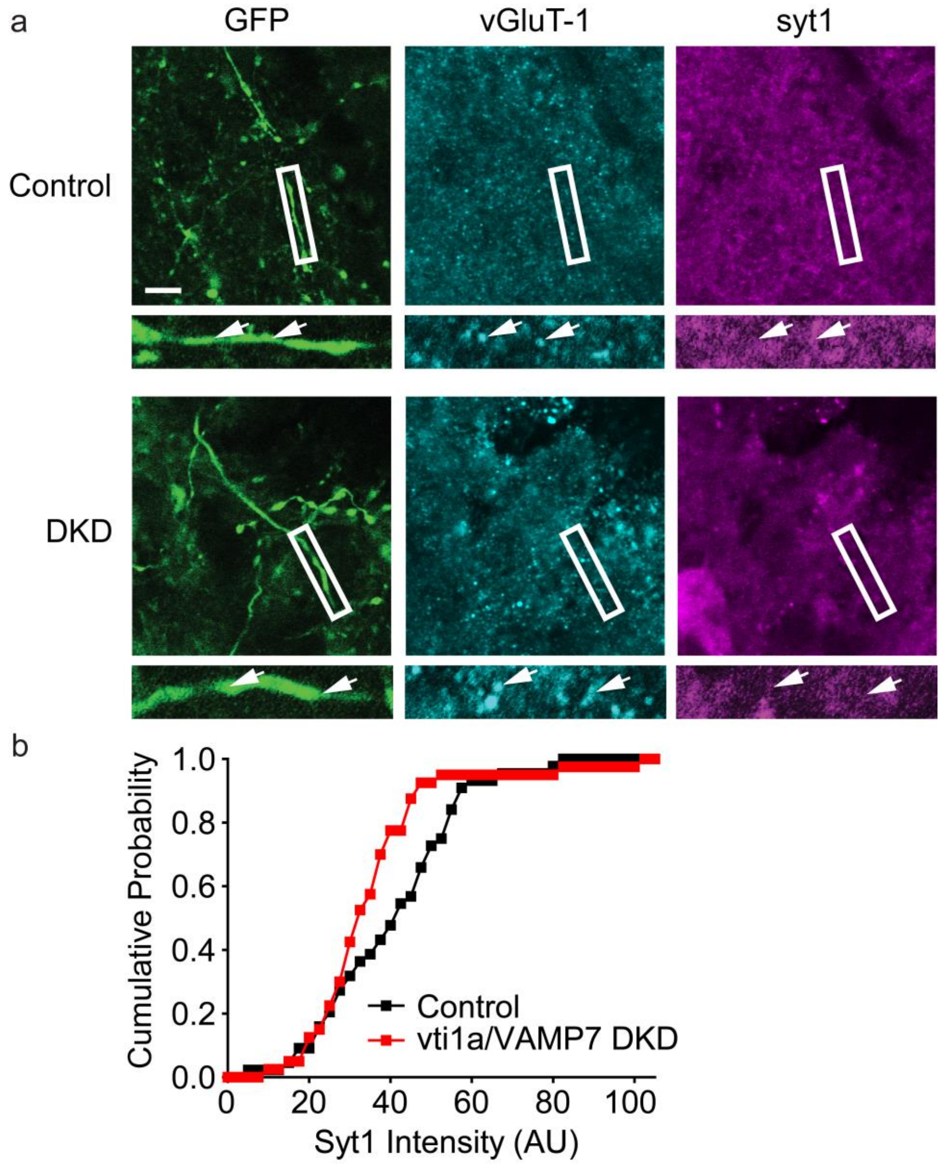

Supplementary Figure 7. Presynaptic loss of vti1a and VAMP7 impairs synaptic vesicle trafficking at rest in mouse hippocampal slices.

a. Representative images of anti-luminal domain of synaptotagmin 1 antibody (syt1) uptake at rest in mossy fiber axons in CA3 *stratum lucidum* expressing GFP (control) or vti1a/VAMP7 DKD. Vesicular glutamate transporter 1 (vGluT-1) immunostaining was used to identify glutamatergic synapses along GFP-positive mossy fiber axons. Scale bar represents 20 μm. Insets: white arrows indicate representative areas selected for syt1 intensity analysis typical of excitatory synapses within infected axons. **b.** Cumulative probability histogram of background-subtracted syt1 antibody intensity at rest in

control and vti1a/VAMP7 DKD mossy fiber axons (control: n=44 slices from 11 mice; vti1a/VAMP7 DKD: n=40 slices from 10 mice). Syt1 intensity is significantly decreased in vti1a/VAMP7 DKD axons compared to control axons (Kolmogorov-Smirnov test; p=0.01).

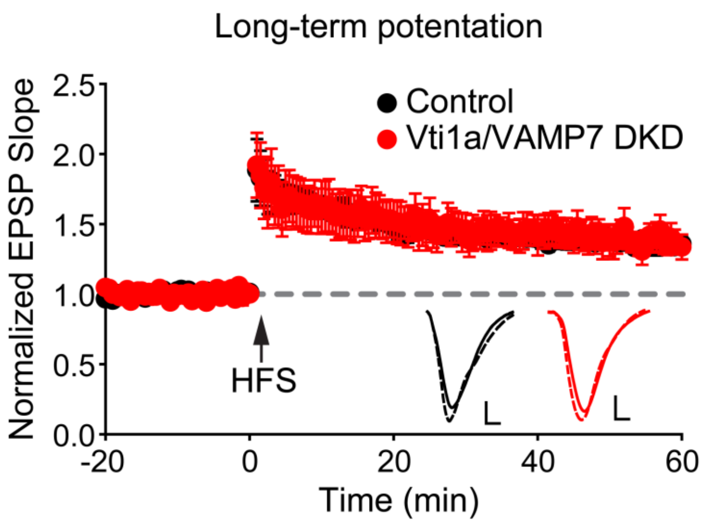

Supplementary Figure 8. Presynaptic loss of vti1a and VAMP7 does not alter high frequency stimulation-induced long-term potentiation in hippocampal slices.

Long-term potentiation (LTP) was not altered in Schaffer collateral synapses after GFP (control) or vti1a/VAMP7 DKD virus was delivered to area CA3 (control: n=13 slices from 4 mice; vti1a/VAMP7 DKD: n=6 slices from 3 mice; p=0.77). Arrow indicates when high frequency stimulation (HFS) was applied to Schaffer collateral axons. Inset: representative traces showing increased response (dashed line) after LTP induction. Scales are 0.3 mV (control) and 0.6 mV (vti1a/VAMP7 DKD) by 2 ms.

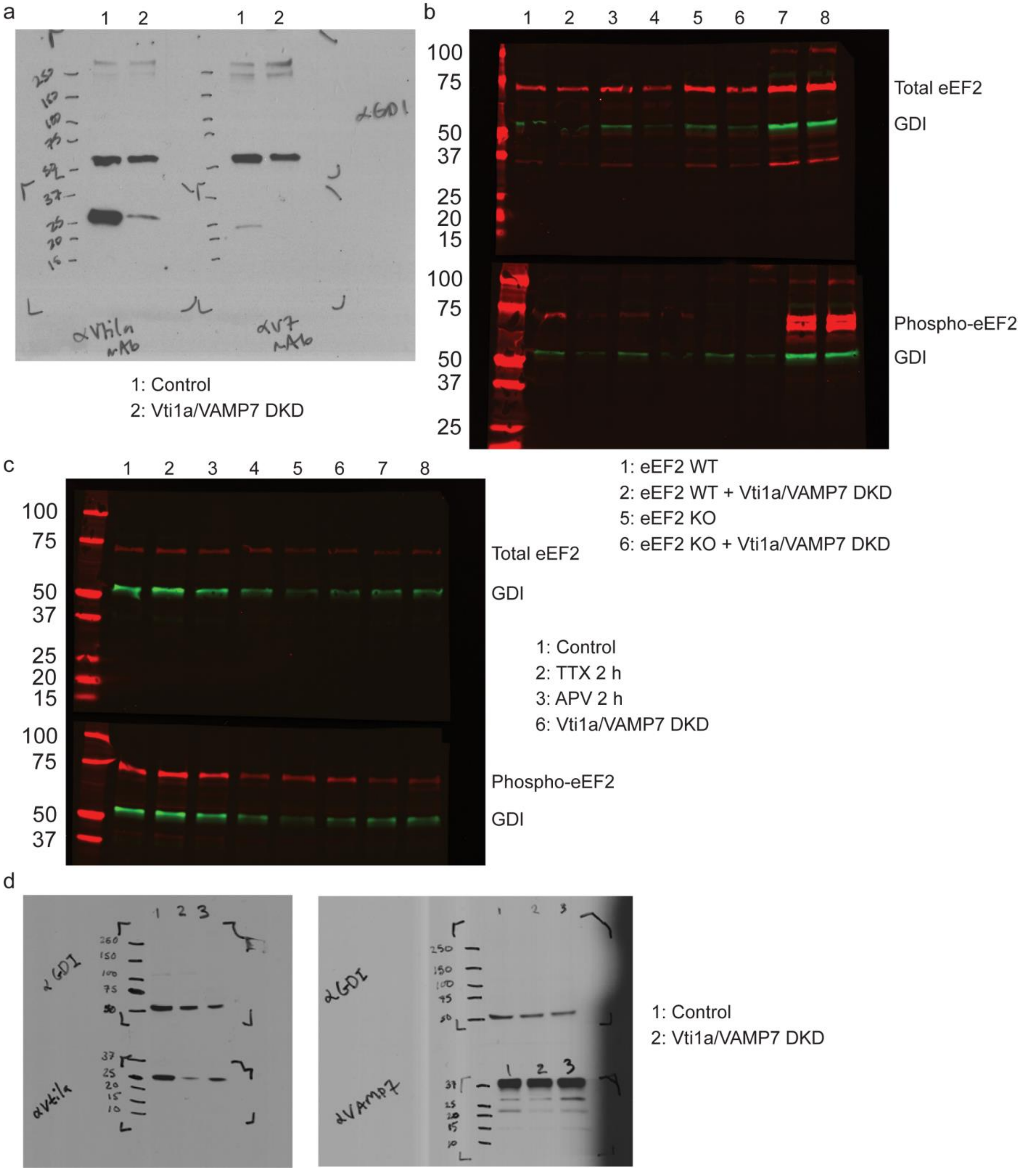

Supplementary Figure 9. Uncropped scans of Western blot images. a. Full scans of Western blot images used in Supplementary Figure 1. **b.** Full scan of Western blot image used in Figure 4. **c.** Full

scan of Western blot image used in Supplementary Figure 4. **d.** Full scans of Western blot images used in Supplementary Figure 6.
